# Propensity to trust in Large Language Models

**DOI:** 10.1371/journal.pone.0347328

**Published:** 2026-05-06

**Authors:** Alice Plebe

**Affiliations:** Department of Industrial Engineering, University of Trento, Trento, Italy; University of Exeter, UNITED KINGDOM OF GREAT BRITAIN AND NORTHERN IRELAND

## Abstract

Trust is central to collaborative settings in which large language models (LLMs) are increasingly deployed. Yet little is known about whether LLMs exhibit a *propensity to trust* (PTT): a baseline tendency to extend or withhold trust that remains relatively stable across contexts. We investigate PTT in nineteen LLMs using two complementary approaches: a psychological self-report scale adapted from human research and a linguistic simulation framework designed to elicit trust-related decisions in context. While the questionnaire produces uniformly high PTT across models—likely reflecting social-alignment objectives and sycophantic response patterns—the simulation framework uncovers substantial, systematic differences in how models entrust others. Our simulations show that trust behavior is governed by the interaction between a baseline tendency to delegate and a model’s capacity to integrate cues about trustworthiness. More capable models, such as GPT-4o-mini, use such cues to adjust their decisions, allowing competence signals to modulate baseline tendencies. By contrast, other models, such as Llama-2-7B, exhibit stable delegation patterns that are largely insensitive to task-specific evidence, leading to systematic over-entrustment. These results show that performance depends not on baseline tendencies alone, but on how they are modulated by alignment-sensitive information. Ablation studies show that task-specific memory mechanisms enable models to better integrate trustworthiness cues, improving the calibration of delegation decisions. More generally, our findings show that questionnaire-based measures cannot disentangle baseline tendencies from context-sensitive adjustment, whereas behavioral simulations make this distinction observable.

## 1. Introduction

Large language models (LLMs) are increasingly assuming roles in social and collaborative environments that were once the exclusive domain of humans [1–8]. To cooperate effectively—whether with humans or other artificial agents—an entity must display key elements of social cognition. Among these, trust stands as a fundamental pillar: it enables agents to predict others’ behavior, coordinate decisions, and sustain collaboration [9–13].

As LLMs increasingly participate in activities that rely on social coordination, the question naturally arises: are they able to trust? Understanding these delegation patterns is essential if LLMs are to function as reliable collaborators. In human psychology, one construct used to describe stable differences in reliance behavior is the *propensity to trust* (PTT): a baseline tendency to grant or withhold trust that does not depend on the immediate situation or the specific trustee [14–17].

Despite its importance, whether LLMs exhibit such stable patterns of trust-related behavior has received limited systematic attention. Existing studies typically operationalize trust as a context-bound variable assessed through classical economic games [18–21]. Although these paradigms are well established for symbolic agents, they fail to engage the linguistic reasoning that constitutes the core competence of LLMs. Language is not merely a means of communication; it provides a substrate for social cognition [22], enabling agents to express commitments, evaluate intentions, and negotiate reliability [23,24]. Evaluating PTT in LLMs therefore requires observing them in language-mediated interactions that reflect the settings in which they are deployed.

To address this gap, we introduce a framework for assessing PTT in LLMs through simulated, language-based interactions. We evaluate nineteen models from OpenAI, Anthropic, Meta, Google, and Microsoft across three ecologically grounded scenarios. In each setting, models decide whether to entrust tasks to specific agents and update their beliefs about each agent’s trustworthiness based on linguistic feedback.

We also administer a human PTT questionnaire [25] as a complementary measure. However, because PTT is framed as an ethically positive trait, socially aligned LLMs may be predisposed to overstate it, raising the question of whether direct self-reports can meaningfully capture trust-related tendencies in these systems.

Importantly, applying the notion of PTT to LLMs does not imply that these systems possess human-like social dispositions. In this work, PTT is used as an operational construct describing stable patterns in delegation behavior across contexts. The focus is therefore not on whether models possess a psychological trait in a human sense, but on whether their decisions exhibit consistent baseline tendencies across scenarios and how these interact with evidence about trustworthiness. This allows systematic comparison of trust-related behavior while remaining agnostic about underlying mental states.

Our results reveal three core findings:

1. Questionnaire-based PTT is not predictive of observed delegation behavior. Social alignment drives models to endorse prosocial statements, producing uniformly inflated scores that mask meaningful differences in how models allocate trust.
2. When evaluated in language-mediated interaction, models show substantial and systematic divergence in their enacted PTT. Some models, most notably Llama-2-7B, trust generously across all settings despite reporting low PTT in the questionnaire. Others, such as Qwen2.5-7B, display the opposite pattern: they report high PTT yet behave cautiously in the simulations.
3. Trust behavior reflects the interaction between baseline delegation tendencies and sensitivity to trustworthiness cues. Access to task-specific memory enables models to modulate their baseline inclination to trust when presented with evidence about a trustee’s competence.

Taken together, these contributions establish a linguistically grounded framework for studying trust in LLMs and highlight the need for behavioral methods that capture how these systems reason about and interact with others. As LLMs become integrated into collaborative settings, understanding how models calibrate trust in others becomes essential for their safe and effective deployment.

## 2. Theoretical background

Trust is widely recognized as a central mechanism enabling cooperation under uncertainty. Despite the intuitive familiarity of the concept, its theoretical foundations vary substantially across disciplines, each emphasizing different aspects of the phenomenon. This section reviews these traditions in order to situate our operational framework within the broader landscape of trust research and to clarify which dimensions of trust are adopted, abstracted, or deliberately excluded in this study.

### 2.1. Trust across disciplines

Compared with many other aspects of social cognition, trust received relatively limited attention in early Western philosophy, with notable discussions appearing in the works of Hobbes, Locke, and Hume [26–28]. Systematic research on trust emerged only in the late twentieth century and has since expanded across multiple disciplines, including philosophy [29–33], psychology [34–38], sociology [39–43], economics [44–49], cognitive science [50], organizational research [51,52], and neuroscience [53–56].

Philosophical research on trust has addressed both conceptual and normative questions concerning the nature of trust and its role in interpersonal relations. A central issue concerns the mental attitude involved in trusting another agent: some accounts interpret trust primarily as a form of expectation or belief about another’s behavior, while others emphasize its distinctive normative structure. Within contemporary discussions, several influential theories are often grouped under the label *motives-based* accounts [32]. According to these views, trust involves assumptions about the trustee’s motivations to fulfill the trust relationship. For example, [29] and [31] argue that trust depends on expectations about the trustee’s reasons or incentives for acting in the trustor’s interest. Other philosophers emphasize the trustor’s reactive attitudes rather than the trustee’s motivations. On this view, the distinctive feature of trust lies in the normative response that follows when trust is violated. [30], for instance, argues that trust is characterized by the trustor’s sense of betrayal when the trusted party fails to act as expected. These debates illustrate that philosophical analyses of trust often extend beyond predictive expectations to include moral obligations, vulnerability, and the interpersonal norms governing trust relationships.

In sociology, trust is commonly analyzed as a structural feature of social systems. [39] and [40], for example, describe trust as a mechanism that reduces social complexity and enables coordination under uncertainty. Sociological work often intersects with economic perspectives that treat trust as a mechanism facilitating cooperation in markets and organizations [41,44,46–49].

Psychological research approaches trust primarily as a behavioral and cognitive phenomenon rather than a normative one. Early developmental theories linked trust to personality formation and attachment processes [34,57]. Subsequent work has explored how trust interacts with learning, interpersonal relationships, and social expectations [36,58–60]. This tradition also overlaps with related fields. Neuroscientific research investigates the neural mechanisms underlying trust-related decisions [53–56], while comparative cognition examines trust-like behaviors in non-human animals—an area where the literature remains scattered [61], despite clear evidence that basic forms of trust play a significant role in social animals, particularly in reciprocal behaviors.

In research on social cognition and language, trust is often examined in relation to the communicative mechanisms that support cooperation. Language allows agents to make explicit commitments, communicate intentions, and reason about the trustworthiness of others. Developmental accounts of human cooperation emphasize the role of linguistic communication in the emergence of shared intentionality and coordinated social behavior [22]. In this perspective, language provides a medium through which agents form, revise, and communicate expectations about trustworthiness [23,24].

### 2.2. Dimensions of trust

Cognitive science and organizational research have sought to formalize these insights in computational or decision-theoretic models of trust. Such work attempts to identify a set of dimensions that characterize how agents evaluate potential collaborators, providing conceptual tools that can be adapted for the analysis of artificial agents.

Among the most influential multidimensional accounts is the organizational framework proposed by [51], which identifies three characteristics of the trustee that shape trust: *ability*, *benevolence*, and *integrity*. Ability refers to the skills or competencies enabling effective action within a domain; benevolence denotes a willingness to act in the trustor’s interest; and integrity concerns adherence to principles that the trustor finds acceptable. A meta-analysis of 132 studies confirmed the empirical robustness of this dimensional approach across a variety of organizational contexts [14].

Computational approaches to trust in cognitive science have proposed related models. [50], for instance, distinguish among several components of trust, including *competence*, *predictability*, and *willingness*. Competence refers to the capacity to perform a task successfully; predictability concerns the consistency with which an agent behaves as expected; and willingness captures the agent’s commitment to carrying out the relevant actions.

Other research on trust in artificial systems adopts similar dimensional frameworks. For example, [62] identify *capability*, *reliability*, *sincerity*, and *ethics* as determinants of human trust in robots, while [63] highlight *competence*, *predictability*, *willingness*, and *honesty* as central elements underlying trust in artificial intelligence systems. Across these approaches, trustworthiness is commonly analyzed in terms of an agent’s competence or capability, the reliability or predictability of its behavior, and its motivational orientation toward fulfilling commitments. Table 1 summarizes the principal proposed trust dimensions.

**Table 1:** Dimensions of trusts in the literature.

Source	Dimensions of trust			
Mayer et al. (1995) [51]	Ability	–	Benevolence	Integrity
Castelfranchi & Falcone (2010) [50]	Competence	Predictability	Willingness	–
Ullman & Malle (2018) [62]	Capability	Reliability	Sincerity	Ethic
Lewis & Marsh (2022) [63]	Competence	Predictability	Willingness	Honesty
**This study**	**Capability**	**Reliability**	**Willingness**	**–**

Examples of theoretical frameworks that analyze trust in terms of dimensions of trustworthiness. These approaches attempt to identify the attributes of an agent that justify reliance in cooperative contexts.

### 2.3. Propensity to trust

While the dimensions discussed above characterize the attributes of a potential trustee, individuals differ considerably in how they interpret and respond to those attributes. Faced with comparable evidence regarding another agent’s trustworthiness, some individuals readily choose to rely on others whereas others remain cautious.

Such interindividual variation is captured by the construct of *propensity to trust* (PTT): a stable individual difference in the baseline likelihood of choosing to rely on another agent when presented with comparable evidence regarding their trustworthiness. The concept first appeared in mid-twentieth-century psychology. [34] suggested that a basic tendency to trust develops during early childhood as part of personality formation. Later work framed this disposition as a stable individual difference influencing trust-related behavior in adulthood [36,37]. Within organizational research [51], formalized the concept of *propensity to trust* as a general willingness to rely on others independent of specific situational cues. Subsequent studies have linked PTT to broader attitudes toward risk-taking and cooperation in professional settings [14,64]. Neuroscientific evidence further suggests that individual differences in trust propensity are associated with neural activity in brain networks involved in social cognition and decision-making [65].

A substantial strand of research concerns the measurement of trust propensity. Early approaches relied on general trust questionnaires [36,64,66], but these instruments often conflated dispositional trust with judgments about specific targets. More recent work has therefore developed dedicated measurement scales designed explicitly to capture PTT [15,17,25,67–69]. A meta-analysis of 179 studies identified 27 distinct instruments used to measure trust propensity across the literature [16]. Among the most widely adopted is the scale proposed by [25], which reduced an initial set of 43 items to a concise 12-item measure. The items of this scale are reported in Table 2.

**Table 2:** Human PTT measurement scale.

1.	It is easy for me to trust others.
2.	Even if I am uncertain, I will generally give others the benefit of the doubt.
3.	I generally believe that others can be counted on to do what they say they will do.
4.	**I usually trust people until they give me a reason not to trust them.**
5.	I tend to trust others even if I have little knowledge of them.
6.	I generally give people the benefit of the doubt when I first meet them.
7.	**Trusting another person is not difficult for me.**
8.	**My typical approach is to trust new acquaintances until they prove I should not trust them.**
9.	I am seldom wary of others.
10.	I don’t mind giving up control to others over matters which are essential to my future plans.
11.	I believe that people usually keep their promises.
12.	**My tendency to trust others is high.**

Items from the Frazier et al.’s *propensity to trust* scale [25]. The four statements with the highest factor loadings are shown in bold.

### 2.4. Trust and LLMs

Research on trust in artificial agents has primarily examined human trust in technology. This literature investigates when and why people rely on AI systems, robots, or automated decision tools [70–80].

More recently, studies have begun to explore trust-related behaviors exhibited by artificial agents themselves. [81] analyze multi-agent systems composed of LLMs from a robustness and security perspective, showing that LLM agents often treat peer-generated content as uniformly credible unless skepticism is explicitly induced. As a result, such systems may become vulnerable to misinformation, manipulation, or coordination failures.

Another line of research examines trust-related behavior in LLMs using experimental paradigms from behavioral economics. [82], for example, study LLM behavior in the classical Trust Game and report that advanced models display patterns resembling human trust decisions, adjusting their behavior in response to perceived risk and potential reciprocity. While such studies demonstrate that LLMs can reproduce recognizable patterns of trust behavior, the analysis is typically restricted to a single, highly simplified decision problem.

Economic trust games provide a well-established experimental paradigm, but they capture only a narrow aspect of trust-related decision making. In these settings, trust is expressed through a small set of numerical choices within a fixed payoff structure. As a result, the agent’s decision depends primarily on quantitative parameters such as risk and expected reciprocity.

The approach adopted here instead focuses on trust expressed through natural-language interaction. This choice does not rest on the assumption that all linguistic processing constitutes social cognition, and the ability of LLMs to process language does not in itself imply that they engage in social cognition. Rather, the methodological motivation is tied to the types of interactions in which LLMs are typically deployed. In collaborative settings, trust is often expressed and negotiated through communicative acts such as requests, commitments, explanations, and feedback.

Language-mediated scenarios therefore provide a context in which models must interpret task descriptions, evaluate information about collaborators, and revise expectations based on textual evidence. Such settings allow multiple trust-relevant cues to be presented and integrated over the course of interaction, making it possible to observe how models respond to richer contextual information about potential collaborators.

Moreover, the construct validity of economic trust games remains debated, with some analyses suggesting that they may conflate trust with related constructs such as risk preference or expectations of reciprocity [83]. Without taking a position on this debate, this observation highlights the value of employing complementary methodologies when studying trust-related behavior in artificial agents. Natural-language simulations provide one such complementary approach, allowing trust to be examined in interactional contexts that more closely resemble the communicative environments in which LLMs typically operate.

### 2.5. Conceptual framework of the present study

The present study examines a functional component of trust widely discussed in psychology, economics, and socio-cognitive modeling: the decision to rely on another agent when the outcome of an action depends on that agent’s behavior under uncertainty. Rather than studying human trust in artificial systems, we investigate how artificial agents themselves express trust and whether large language models display stable patterns of reliance when delegating tasks to other agents.

Trustworthiness is operationalized using three dimensions that recur across several empirical and computational models: *capability*, *reliability*, and *willingness*. Capability refers to the ability to perform a delegated task successfully. Reliability captures the consistency with which an agent performs successfully across situations. Willingness describes the disposition to carry out the relevant actions rather than neglect or abandon them. The term *willingness* corresponds to the action-oriented component of Mayer et al.’s notion of benevolence. Mayer et al. define benevolence as “the extent to which a trustee is believed to want to do good to the trustor, aside from an egocentric profit motive” [51, p. 718] and further suggest that it may involve a specific attachment to the trustor. In the present framework, we abstract from affective attachment, altruistic motivation, and moral concern, retaining only the behavioral component relevant for task execution.

These three dimensions are not intended to exhaust all theoretical accounts of trust. Philosophical and sociological approaches often incorporate additional normative and relational elements, including moral expectations and reactive attitudes such as resentment or betrayal [30,71]. Our choice is methodological. Capability, reliability, and willingness constitute a minimal, behaviorally operationalizable subset that recurs across influential empirical and computational models of trust (e.g., [51]; [50]). The aim of the present study is not to adjudicate between competing theories of trust, nor to reproduce the full normative richness of human interpersonal trust. Instead, we isolate this widely shared functional core in order to examine whether large language models exhibit stable patterns of reliance across situations, captured by the construct of *propensity to trust*.

PTT is interpreted as a stable difference across agents in the baseline likelihood of relying on another agent when presented with comparable evidence regarding these dimensions. In the context of our simulation, PTT is defined operationally as the stability of delegation decisions across heterogeneous scenarios.

This interpretation does not presuppose that LLMs possess an interior mental life or stable dispositional attitudes in a human-like sense—an assumption that would situate the analysis within ongoing debates about machine mentality and selfhood [84–86]. The present work remains agnostic on these questions. Whether LLMs possess genuine mental states is a substantive philosophical question, but resolving it is not required for the present analysis, which focuses on observable behavioral regularities in model outputs rather than on claims about internal psychological states.

Accordingly, trust-related constructs are treated here as abstractions summarizing observable behavioral regularities in model outputs rather than as claims about an underlying mental ontology. In this respect, the framework follows what [87] describe as *anthropocentric abstraction*: employing conceptual frameworks originating in human social cognition at a level of abstraction that preserves their functional role while remaining neutral about their metaphysical interpretation.

## 3. Methodology

This study evaluates LLMs’ scenario-independent tendency to delegate tasks under uncertainty through two complementary approaches: direct responses to standardized human PTT questionnaires and behavioral observation in simulated collaborative scenarios.

In the questionnaire-based evaluation, we measure each model’s self-reported PTT using the 12-item scale developed by [25] (Table 2), one of the most comprehensive and linguistically refined instruments for assessing human trust disposition. Each item expresses a trust-related attitude and requires a response on a seven-point Likert scale ranging from complete disagreement to complete agreement.

In the simulation-based evaluation, we employ a task assignment setting that elicits trust-related decisions through natural language interaction. This approach provides an indirect measure of PTT by observing how each model decides whether to entrust specific agents with a task, updates its beliefs about their trustworthiness, and adjusts subsequent choices accordingly. Unlike questionnaire-based assessment, the simulation does not rely on self-reporting; instead, it captures behavioral consistency across contexts, revealing whether a model exhibits a stable dispositional tendency to trust or to withhold trust.

### 3.1. Task assignment simulation

**Fig 1:**
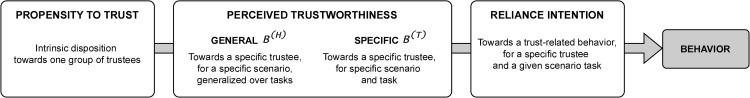
Conceptual model of the trust formation process. Adapted from [68]. In our setting, this process results in a behavioral decision in which the LLM evaluates whether to trust a potential trustee for a given task.

We ground our simulation in the human trust formation process described by [68]. As shown in Fig 1, a model’s PTT represents its baseline tendency to delegate across a broad class of potential trustees. This is the most general level of trust, which becomes increasingly specific as the model forms beliefs about particular candidates. For each trustee, the model develops two forms of perceived trustworthiness: general, applying across tasks within a scenario, and task-specific, applying to a particular assignment. These beliefs inform the model’s intention to rely on a given trustee when deciding whether to delegate a task. The process culminates in a trust-related behavior, expressed as the model’s decision to assign—or not assign—the task to that trustee.

We represent the trustees as a team 𝒜 of agents, where each agent is defined as:


a=(n,𝐱), (1)

with $n\in A^{*}$ denoting the agent’s name (where *A* is the set of alphanumeric characters and A^*^ the set of all possible strings), and $𝐱\in {\mathbb{R}}^{3}$ encoding the agent’s internal properties along the trust dimensions of *capability*, *reliability*, and *willingness*. These properties are hidden from all other agents and from the trustor.

We define a task *t* as:


t=(d,𝐲), (2)

where $d\in A^{*}$ is the textual description of the task, and $𝐲\in {\mathbb{N}}^{3}$ specifies the required levels of *capability*, *reliability*, and *willingness* for successful completion. Task requirements are also hidden from the trustor, who must infer them from the textual description *d*. In the simulation, an agent *a* may successfully complete a task *t* depending on how closely its property vector **x** aligns with the task requirements **y**. The algori*t*hm used to evaluate this alignment is described in Section 3.1.1.

The trustor, represented by the LLM, is equipped with a short-term memory *S*:


$$S=\left(\left\{B_{i}^{\left(H\right)}\right\},\left\{B_{i,j}^{\left(T\right)}\right\}\right),$$ (3)

which stores the perceived trustworthiness of the trustees, expressed in natural language. The two belief components differ in their level of specificity: $B_{i}^{\left(H\right)}$ encodes the trustor’s general assessment of trustee *a*_*i*_, whereas $B_{i,j}^{\left(T\right)}$ captures expectations about *a*_*i*_’s performance on a particular task *t*_*j*_. Both beliefs are updated over the course of the simulation based on the trustor’s observations. $B_{i}^{\left(H\right)}$ consists of a linguistic statement summarizing the trustor’s overall view of *a*_*i*_ within the scenario and is revised after every decision to delegate a task to that agent. By contrast, $B_{i,j}^{\left(T\right)}$ is task-specific and is revised only when the trustor assigns task *t*_*j*_ to *a*_*i*_.

The simulation progresses through a sequence of events, each following six steps:

1. The system randomly selects a task *t*_*j*_ from the scenario.
2. The system selects an agent *a*_*i*_ from the team 𝒜 in cyclic order.
3. The trustor decides whether to entrust the task *t*_*j*_ to agent *a*_*i*_, based on the task description *d*_*j*_ and its current beliefs $B_{i}^{\left(H\right)}$ and $B_{i,j}^{\left(T\right)}$.
4. If the decision is negative, the simulation returns to step 2. If it is positive, the system computes the alignment between the trustee’s properties **x**_*i*_ and the task requirements **y**_*j*_ to determine the task outcome (see Section 3.1.1 for details).
5. The trustor receives a message *o*_*D*_ summarizing the task outcome. In cases of success, this is a general statement; in cases of failure, it is an indirect linguistic description of the dimension of **x**_*i*_ that caused the failure.
6. The trustor updates its trust beliefs about *a*_*i*_ based on *o*_*D*_. It retrieves the most recent $B_{i}^{\left(H\right)}$ and $B_{i,j}^{\left(T\right)}$, revises them according to the outcome, and stores the updated beliefs in short-term memory.

At the beginning of the simulation, $B_{i}^{\left(H\right)}$ and $B_{i,j}^{\left(T\right)}$ are empty. To initialize the trust formation process, the system performs a bootstrapping round that provides the trustor with preliminary beliefs about each trustee. Before the first event, the system parses all possible task—agent combinations. During this phase, the trustor does not make decisions but observes outcomes, forming an initial impression of each agent’s trustworthiness across tasks.

#### 3.1.1. Computation of task–agent alignment.

The outcome of a task *t* executed by agent *a* is modeled probabilistically as a function of how closely the agent’s properties **x** align with the task requirements **y**. Alignment is evaluated holistically: strong values in some trust dimensions can compensate for weaker values in others.

Let $o_{B}\in \{0,1\}$ denote the outcome of the task, where *o*_*B*_ = 1 indicates successful completion and *o*_*B*_ = 0 indicates failure. The probability of success, $p(o_{B}=1|𝐱,𝐲)$, depends on the overall match between **x** and **y**, measured by their dot product:


$$R=𝐲\cdot {𝐱}^{⊤}.$$ (4)

This score *R* quantifies how well an agent’s attributes match the task requirements and serves as the basis for computing the corresponding probability of success. To generate distinct alignment scores for each possible agent–task pairing, we consider six agent profiles **x** given by permutations of [0,1,2] and six task profiles **y** given by permutations of [1,2,4].

The resulting alignment scores partition agent–task combinations into two groups (Table 3): high-ranking alignments associated with a high probability of success $p(o_{B}=1)\gg 0.5$, and low-ranking alignments associated with a low probability of success $p(o_{B}=1)\ll 0.5$. The boundary between these groups is controlled by a difficulty parameter η∈ℕ. A small stochastic component r∈ℝ introduces randomness into the outcome.

**Table 3:** Example of task–agent alignment computation.

y	x	$R=𝐲\cdot {𝐱}^{⊤}$	*p*(*o*_*B*_ = 1)				
			η=4	η=3	η=2	η=1	η=0
$\left[\begin{array}{c} 1,2,4 \end{array}\right]$	$\left[\begin{array}{c} 0,1,2 \end{array}\right]$	10	1 – *r*	1 – *r*	1 – *r*	1 – *r*	1 – *r*
$\left[\begin{array}{c} 1,2,4 \end{array}\right]$	$\left[\begin{array}{c} 1,0,2 \end{array}\right]$	9	**5** ***r***	1 – 2*r*	1 – 2*r*	1 – 2*r*	1 – 2*r*
$\left[\begin{array}{c} 1,2,4 \end{array}\right]$	$\left[\begin{array}{c} 0,2,1 \end{array}\right]$	8	**4** ***r***	**4** ***r***	1 – 3*r*	1 – 3*r*	1 – 3*r*
$\left[\begin{array}{c} 1,2,4 \end{array}\right]$	$\left[\begin{array}{c} 2,0,1 \end{array}\right]$	6	**3** ***r***	**3** ***r***	**3** ***r***	1 – 4*r*	1 – 4*r*
$\left[\begin{array}{c} 1,2,4 \end{array}\right]$	$\left[\begin{array}{c} 1,2,0 \end{array}\right]$	5	**2** ***r***	**2** ***r***	**2** ***r***	**2** ***r***	1 – 5*r*
$\left[\begin{array}{c} 1,2,4 \end{array}\right]$	$\left[\begin{array}{c} 2,1,0 \end{array}\right]$	4	***r***	***r***	***r***	***r***	***r***

The table illustrates how the alignment between a task’s requirements (**y**) and a trustee’s properties (**x**) determines the success probability, which varies with simulation difficulty (η). Combinations with a higher likelihood of success are highlighted in green, while those more likely to result in failure are shown in red. A small component *r* introduces stochasticity in the outcome.

The three dimensions of agent properties enter symmetrically into the task–agent alignment mechanism. No dimension is privileged a priori, and any of them may determine success depending on the task. The alignment computation therefore evaluates multi-dimensional fit rather than a single dominant trait. The dot-product formulation allows partial compensation across the three dimensions while still favoring alignment with the highest-weighted components of **y**. This is a modeling choice that provides a simple and continuous measure of task–agent compatibility, rather than a theoretical claim about the nature of trust. Alternative non-compensatory formulations (e.g., threshold or conjunctive rules) could also be explored.

Importantly, the vector representations of agent properties and task requirements are not accessible to the LLM. The model receives only natural-language task descriptions and outcome feedback, while alignment is computed probabilistically in the background. From the model’s perspective, the task consists solely of interpreting linguistic cues and updating beliefs based on textual outcomes, rather than solving an explicit matching problem.

If delegation behavior were driven by an implicit matching strategy, it would be expected to converge toward optimal assignment. Instead, we observe systematic differences in delegation patterns across models, including persistent over- and under-entrustment (Section 5). This indicates that decisions reflect both evidence integration and model-specific baseline tendencies, rather than purely logical problem solving.

## 4. Evaluation setup

We evaluate 19 large language models for their propensity to trust. The selection spans major contemporary model families developed by OpenAI, Anthropic, Meta, Google, and Microsoft, as well as open-weight releases from Mistral and Alibaba’s Qwen initiatives. Table 4 lists all evaluated models, reporting both their full names and the short codes used used for brevity in subsequent tables and figures.

**Table 4:** Evaluated LLMs.

GPT-3.5-turbo	(gpt35)	Phi-3-mini-4K-instruct	(ph3m)
GPT-4	(gpt4)	Qwen1.5-7B-Chat	(qw1–7)
GPT-4o	(gpt4o)	Qwen2.5-7B-Instruct	(qw2–7)
GPT-4o-mini	(gpt4om)	Qwen2.5-14B-Instruct-1M	(qw2–14)
GPT-4.1-mini	(gpt41m)	Claude-3-haiku-20240307	(cl3h)
GPT-oss-20B	(gptoss)	Claude-3.5-haiku-20241022	(cl3.5h)
Gemma-2-9B-it	(gem2–9)	Claude-3.5-sonnet-20240620	(cl3.5s)
Llama-2-7B-chat-hf	(ll2–7)	Claude-3.7-sonnet-20250219	(cl3.7s)
Llama-2-13B-chat-hf	(ll2–13)	Claude-3-opus-20240229	(cl3o)
Llama-3.1-8B-Instruct	(ll3–8)		

Evaluated LLMs and short codes used for brevity in subsequent figures.

### 4.1. Questionnaire-based evaluation

We administer the 12 items by [25] (Table 2) to the 19 LLMs under investigation. We ask models to respond on a seven-point Likert scale ranging from *complete disagreement* to *complete agreement*. To account for response variability, we present each item 10 times to every model and average the results across repetitions.

### 4.2. Simulation-based evaluation

We evaluate the LLMs’ trust propensity across three distinct scenarios: responding to a building fire, where agents perform firefighting and first-aid tasks (fire); maintaining a farm, where agents carry out agricultural and mechanical tasks (farm); and managing a school, where agents handle administrative and organizational tasks (school). See S1 Appendix for further details.

Each scenario includes six possible tasks, each associated with a different requirement vector **y**, that is one of the six permutations described in Section 3.1.1. The model (trustor) interacts with a team of six agents (trustees), each defined by a distinct property vector, also drawn from the six permutations described in Section 3.1.1.

Simulations unfold entirely through text-based interaction: tasks, agent behaviors, and outcomes are all represented linguistically, and the model’s trust-related decisions take place exclusively in natural language. The three scenarios differ in narrative framing, task structure, urgency, and required competencies, while sharing only minimal vocabulary overlap. This diversity prevents models from relying on superficial lexical cues and instead reveals their broader disposition toward trusting others across heterogeneous linguistic contexts.

In addition, we test two ablation settings of each of the 19 LLMs. The first (1-mem) retains only general perceived trustworthiness and excludes task-specific beliefs, such that Equation (3) reduces to $S=\left\{B_{i}^{\left(H\right)}\right\}$. The second (no-trust) also uses a single memory and, in addition, removes any explicit mention of trust by excluding the capability, reliability, and willingness dimensions from the prompts. See S2 Appendix for examples of prompts used in each case.

Each simulation runs for 50 events, and we repeat 10 simulations for every combination of model, scenario, and ablation configuration. All runs use a medium difficulty level (η=2) and include a small stochastic component (*r* = 0.01).

## 5. Results

### 5.1. Results from human questionnaire

**Fig 2:**
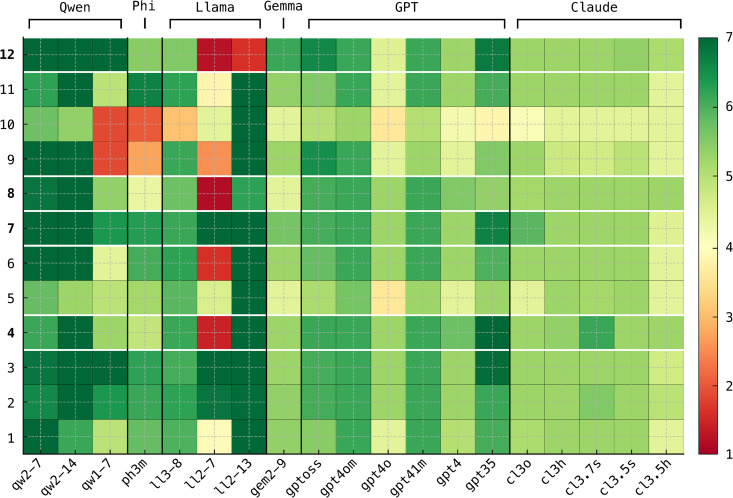
Questionnaire results. Results of the PTT scale from [25] administered to all models. The 12 items from Frazier’s scale are shown, with the four items with the highest factor loadings highlighted in bold. Responses use a 7-point Likert scale (1 = *strongly disagree*, 7 = *strongly agree*).

Figure 2 shows that, across the 19 evaluated models, the average PTT scores on the Frazier scale (Table 2) fall within a narrow and consistently positive range. This indicates that the models exhibit relatively uniform levels of self-reported trust. The mean score reported for human participants is 5.03 [25, p. 86], which lies near the midpoint of the models’ average values, suggesting that most LLMs report trust levels comparable to—or slightly higher than—human baselines.

Apart from a single outlier, Llama-2-7B (ll2–7), whose scores are markedly lower than those of the other models, responses are highly homogeneous across model families. GPT and Claude models display very similar endorsement patterns across items, while the more recent Qwen models (qw2–7 and qw2–14) rank among the highest-scoring systems. Overall, the questionnaire produces a compressed distribution of scores with relatively limited variation between models.

These questionnaire-based results should be interpreted with caution. Many items in the Frazier scale explicitly describe prosocial or socially desirable attitudes, such as giving others the benefit of the doubt or assuming good intentions. Because modern LLMs are trained to produce cooperative, helpful, and non-antagonistic responses, they are naturally inclined to endorse such statements. As a result, high questionnaire scores may reflect agreement with socially desirable content rather than stable patterns of delegation behavior expressed during interaction.

This pattern can be explained by alignment procedures used in contemporary LLM training. Models fine-tuned with reinforcement learning from human feedback (RLHF) or related methods are optimized to produce responses perceived as helpful, cooperative, and socially appropriate [88,89]. Consequently, they tend to endorse statements expressing socially valued attitudes, a behavior often described as sycophancy [90,91]. When applied to questionnaire-style prompts, this leads to systematically inflated trust scores.

This does not imply that such responses are irrelevant to model behavior. Rather, the limitation is one of measurement: questionnaire responses reflect prompt-level agreement with prosocial statements, whereas our objective is to assess how models allocate trust when confronted with varying evidence about collaborators. Consequently, questionnaire-based measures cannot distinguish between training-driven agreement and consistent delegation behavior across contexts.

### 5.2. Results from task assignment simulations

**Fig 3:**
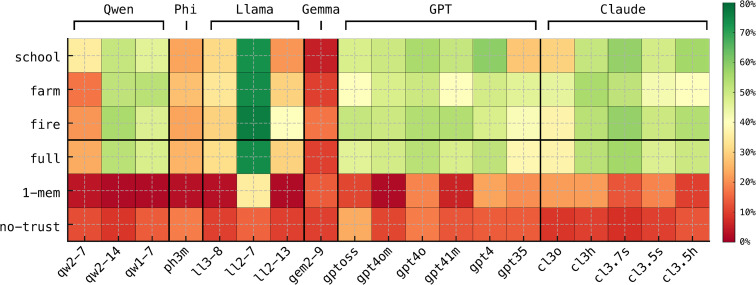
Simulation results. Proportion of task assignments in which a model decides to entrust the selected agent, across scenarios and ablation configurations. The bottom three rows (full, 1-mem, no-trust) aggregate results across all scenarios for the corresponding ablation; the top three rows (school, farm, fire) correspond to the full models.

Fig 3 shows how often each model decides to entrust a task to an agent, across scenarios and ablation configurations. The patterns that emerge differ substantially from the trust tendencies suggested by the questionnaire-based evaluation.

The contrast with Fig 2, which summarizes the questionnaire-based results, is immediate and striking. The model that most frequently chooses to trust agents in the simulations, Llama-2-7B (ll2–7), is the same model that expresses the lowest trust when responding to the questionnaire. A similar discrepancy appears for GPT-4o (gpt4o), which shows a tendency to trust in the simulations but a notably cautious profile in the questionnaire. Conversely, Qwen2.5-7B (qw2–7), which displays high self-reported trust in the questionnaire, shows comparatively low trust levels in the simulation.

This reversal reveals a systematic divergence between simulation behavior and questionnaire responses, indicating that questionnaire-based assessments cannot be used in isolation as a reliable measure of PTT in LLMs.

Additionally, the bottom rows of Fig 3 illustrate the impact of removing components of the trust formation process. The 1-mem ablated variant, which omits task-specific perceived trustworthiness (Fig 1), and the no-trust ablated variant, which removes the three-dimensional definition of trust, both lead to substantially lower rates of trust decisions. These results suggest that task-specific trustworthiness and the structured three-dimensional representation of trust each contribute importantly to the emergence of trust-like behavior in the simulations.

#### 5.2.1. Stability Across Scenarios.

We quantify the stability of each model’s trust behavior across scenarios using several statistical indicators. Our goal is to isolate the extent to which each model exhibits a scenario-independent tendency to trust, i.e., a behavioral signature consistent with a baseline PTT.

Table 5 reports six metrics computed for each model from the fraction of events in which the model entrusts the agent with the task. For each model, we compute:

- $\bar{\mu }$: the overall average entrustment rate across scenarios;
- Δ: the range of entrustment rates;
- σ: the standard deviation across scenarios;
- $\eta^{2}$: the effect size of scenario on trusting decisions, derived via one-way ANOVA (higher values indicate stronger scenario influence);
- ρ: the intra-class correlation coefficient (ICC), measuring the proportion of variance attributable to scenario-specific rather than random effects;
- π: a composite PTT-stability index synthesizing σ and $\eta^{2}$ into a single value in [0,1] (values near 1 indicate strong scenario-independence, and therefore a more baseline dispositional trust tendency).

**Table 5:** Statistics across simulation scenarios.

Model	$\bar{\mu }$	Δ	σ	$\eta^{2}$	ρ	π
gpt35	0.38	0.182	0.093	0.0245	0.034	0.11
gpt4om	0.49	0.020	0.011	0.0003	0.000	1.00
gpt4o	0.53	0.044	0.024	0.0015	0.000	0.90
gpt4	0.52	0.104	0.057	0.0088	0.011	0.59
gptoss	0.46	0.112	0.059	0.0092	0.012	0.57
gpt41m	0.48	0.144	0.076	0.0156	0.021	0.36
cl3h	0.53	0.046	0.023	0.0014	0.000	0.91
cl3o	0.37	0.144	0.072	0.0148	0.020	0.40
cl3.5h	0.50	0.166	0.089	0.0211	0.029	0.20
cl3.5s	0.47	0.082	0.044	0.0053	0.006	0.72
cl3.7s	0.56	0.062	0.036	0.0035	0.112	0.80
ll2–7	0.74	0.030	0.015	0.0008	0.115	0.97
ll2–13	0.30	0.194	0.097	0.0298	0.042	0.00
ll3–8	0.32	0.040	0.023	0.0016	0.000	0.91
qw1–7	0.48	0.074	0.040	0.0042	0.004	0.77
qw2–7	0.24	0.172	0.090	0.0292	0.041	0.05
qw2–14	0.53	0.038	0.021	0.0012	0.000	0.92
gem2–9	0.10	0.124	0.062	0.0278	0.039	0.23
ph3m	0.25	0.038	0.022	0.0017	0.001	0.91

Summary statistics for the fraction of entrustment decisions computed across scenarios. For each model, $\bar{\mu }$ denotes the overall average entrustment rate, Δ the entrustment range across scenarios, and σ the corresponding standard deviation. $\eta^{2}$ reports the scenario effect size from a one-way ANOVA, and ρ the intraclass correlation coefficient. π is the PTT stability index defined in the text.

We define:


$$\pi =w_{\sigma }(1-\sigma )+w_{\eta }(1-\eta^{2})$$ (5)

with $w_{\sigma }=w_{\eta }=0.5$.

Models with the highest π, GPT-4o-mini, Llama-2-7B, Qwen2.5-14B, and Phi-3-mini, show the strongest scenario-invariance, indicating comparatively stable internal PTTs. At the opposite extreme, models such as Llama-2-13B, Claude-3.5-Haiku, and Qwen2.5-7B show pronounced scenario sensitivity, suggesting that their trust decisions depend heavily on scenario content rather than on a consistent underlying disposition.

A core finding is that PTT stability is not correlated with entrusting magnitude. Among the models with highly stable PTTs, we find both Llama-2-7B, the most trusting model overall, and Phi-3-mini, which is among the least trusting. This dissociation confirms that stability and magnitude constitute independent dimensions: a model may be dispositionally trusting, dispositionally distrustful, or display no dispositional pattern at all.

#### 5.2.2. Stability across task-agent alignments.

**Fig 4:**
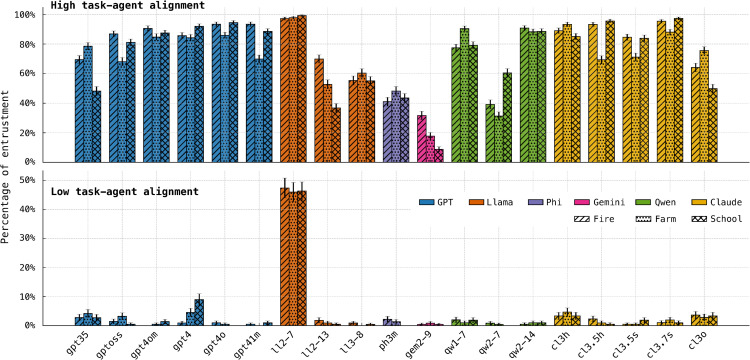
Entrustment decision rates. Percentage of entrustment decisions made by each model across the three simulated scenarios. The top panel reflects high task—agent alignment, where the trustee is well suited to the task; the bottom panel reflects low alignment, where the trustee is poorly matched.

Figure 4 examines how each model’s entrustment decisions vary with task—agent alignment, defined as the match between an agent’s properties and the task’s requirements. The top panel shows cases with high alignment, where the trustee has a high probability of success (*R* > 7, Table 3). Most models behave as expected: they frequently entrust high-alignment agents. GPT-4 models are particularly consistent, with trust rates tightly clustered at high values. By contrast, Gemma-2-9B and Phi-3-mini under-trust even in this favorable setting, maintaining comparatively low entrustment rates despite evidence of trustee competence.

The bottom panel of Fig 4 isolates low-alignment cases, where the trustee is unlikely to complete the task successfully (*R* < 7). Here most models adopt a conservative strategy and rarely entrust low-alignment agents. The most extreme outlier is Llama-2-7B, which entrusts low-alignment agents far more often than any other model. This pattern is stable across all scenarios and is not attributable to noise; it reflects a systematic tendency to extend trust even when the available evidence weighs against it. In contrast, the majority of models behave skeptically in this regime, indicating greater sensitivity to negative evidence.

**Fig 5:**
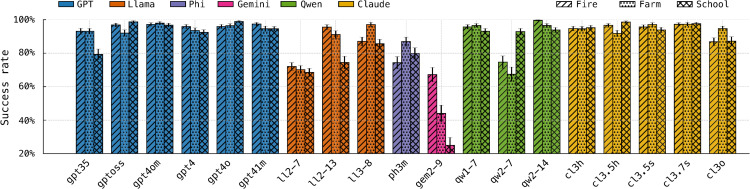
Task completion rates. Percentage of successfully completed tasks by entrusted agents, shown for all models across the three simulated scenarios.

Figure 5 shows the proportion of tasks each model completes successfully across scenarios. Llama-2–7B’s generous entrustment strategy has predictable consequences: assigning tasks to poorly matched agents lowers its success rate. Even so, it still completes around 70% of tasks, indicating that over-trusting behavior is costly but not catastrophic in this setting. By contrast, Gemma-2–9B’s pronounced reluctance to trust often results in no agent being selected for a task, which guarantees failure. This leads to the lowest overall success rate among the models evaluated.

#### 5.2.3. Stability under ablations.

**Fig 6:**
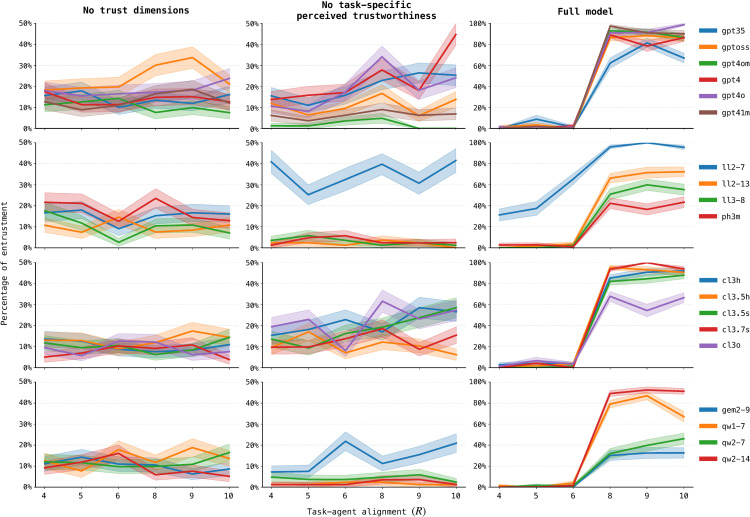
Entrustment rates and ablations. Entrustment decision rates across task–agent alignments for the fire scenario. Columns correspond to different ablation settings: no-trust (left), 1-mem (middle), and full (right). Rows group models by family. Each line shows the percentage of decisions in which a model entrusts the agent at a given alignment level **(*R*)**, with shaded regions indicating variability across runs.

Figure 6 reports entrustment rates across all task—agent alignment levels (*R*, Table 3) for the three ablation conditions introduced in Section 4.2.

Under the no-trust ablation (left column), models lack both trust-related descriptors and task-specific memory, and consequently show little structure in their behavior. Entrustment rates remain low and noisy across alignment levels, indicating that when models receive neither trust cues nor outcome-relevant memory, they cannot form meaningful expectations about agent performance. A few models, most notably GPT-oss-20B, still display weak trends, suggesting that some families encode minimal inductive biases about agent competence even without structured information.

The 1-mem ablation (middle column) produces more differentiated patterns. Removing task-specific beliefs but preserving general perceived trustworthiness allows some models to exploit the limited evidence available: models such as Llama-2-7B, GPT-4, Gemma-2-9B, and Claude-3-opus show a modest increase in entrustment. However, many models behave similarly to the no-trust case. This suggests that a single memory element—general perceived trustworthiness without task-specific structure—provides too weak a signal to support calibrated trust judgments across the full range of alignments.

The full model condition produces a qualitatively different pattern. All models show a clear and systematic increase in entrustment between *R* = 6 and *R* = 8. When both general and task-specific trust beliefs are available, the models become more sensitive to evidence of trustworthiness. The increase is especially pronounced for the OpenAI and Anthropic models, whereas other families, such as Qwen and Gemma, display a more gradual slope.

A further distinction emerges for low-alignment cases (*R* < 7). While most models show near-zero entrustment in this region, Llama-2-7B once again stands out as the only model that entrusts agents at a high rate despite the poor match between agent properties and task requirements. Although Llama-2-7B and Qwen2.5-7B exhibit similarly shaped curves, Qwen2.5–7B’s overall entrustment levels remain much lower, resulting in substantially more cautious trust behavior.

### 5.3. Discussion

Our findings show that trust behavior is governed by the interaction between a baseline tendency to delegate and a model’s capacity to integrate evidence about collaborators. The PTT stability index π, Eq. (5), captures the extent to which delegation rates remain consistent across heterogeneous scenarios. Crucially, π does not measure responsiveness to task–agent alignment (“task–agent alignment” refers to the degree of compatibility between an agent’s properties and a task’s requirements in the simulation (Section 3.1.1), and should not be confused with alignment in the sense of model training, e.g., RLHF): it isolates the scenario-invariant component of behavior, which must be interpreted together with how models react to alignment cues.

The consequences of this interaction are visible when comparing models. Models equipped with a more sophisticated memory mechanism adjust their decisions in response to cues about agent abilities: GPT-4o-mini exemplifies this pattern, showing stable delegation (high π) across scenarios where cues are diffuse, but substantial variation across task–agent alignments, changing behavior strongly depending on cues of competence or clear incompetence. Llama-2-7B exhibits high π but remains largely insensitive to alignment information, continuing to entrust agents even when failure is likely; this produces systematic over-entrustment and reduced performance. Gemma-2-9B shows the opposite pattern, combining low π with consistently low delegation rates and failing to exploit favorable alignments, resulting in systematic under-entrustment. Phi-3-mini occupies an intermediate regime: it combines high π with more conservative delegation rates, avoiding the extreme over-entrustment of Llama-2-7B while maintaining a consistent baseline, and consequently achieves higher overall success than both models. Taken together, these cases show that performance depends not on baseline stability alone, but on how baseline tendencies are modulated by alignment-sensitive evidence.

Differences across model families follow the same pattern. Several commercial models (e.g., the GPT and Claude variants) display strong responsiveness to alignment cues, while earlier open-weight systems such as Llama-2-7B rely more heavily on baseline tendencies. However, this distinction is not fixed: more recent open models (e.g., Qwen2.5-14B and Llama-3.1-8B) show improved calibration, combining more stable baselines with greater sensitivity to competence signals. These trends suggest that trust behavior is shaped by model maturity and training methodology rather than by a structural divide between open and closed systems.

Performance in delegation tasks therefore depends on the joint contribution of baseline PTT and sensitivity to task–agent alignment. Stable baseline tendencies support consistent behavior across contexts, while responsiveness to alignment cues enables adaptation to task-specific evidence; neither component alone is sufficient. Over-entrustment arises when stable baselines are insufficiently modulated by negative evidence, while under-entrustment emerges when weak or unstable baselines are not corrected by positive evidence.

This interaction also exposes a limitation of questionnaire-based PTT measures. Questionnaire responses primarily reflect sycophancy and training-induced endorsement of socially desirable statements [90,91] and do not capture how models balance baseline tendencies with evidence about collaborators. As a result, they cannot distinguish whether observed behavior is driven by stable baseline tendencies, by context-sensitive adjustment, or by their interaction. Llama-2-7B illustrates this mismatch: despite having the lowest questionnaire scores, it exhibits the highest delegation rates in simulation. Behavioral evaluation is therefore necessary to characterize how models allocate trust in context.

PTT itself should be understood as a baseline parameter rather than a directly interpretable indicator of effective trust behavior. Its contribution depends on how it interacts with evidence sensitivity: a stable baseline can support effective delegation, as in Phi-3-mini, but becomes detrimental when not appropriately modulated, as in Llama-2-7B, while weak or unstable baselines lead to persistent under-entrustment, as in Gemma-2-9B. Effective trust behavior emerges from the balance between these two components rather than from either in isolation.

### 5.4. Limitations

Several limitations constrain the scope and interpretation of the present findings. First, the simulated scenarios are restricted to collaborative delegation settings. Trust in adversarial, strategic, high-stakes, or norm-governed environments may involve additional mechanisms not captured here. In particular, contexts involving moral conflict, asymmetric vulnerability, or institutional accountability could alter delegation behavior beyond the *capability–reliability–willingness* framework adopted in this study. Extending the analysis to such settings remains an important direction for future work.

Second, the simulation relies on structured task–agent alignments and relatively transparent feedback. Outcomes are binary (success or failure), and feedback indirectly reveals the dimension associated with failure. Real-world collaboration is typically less informative: feedback may be delayed, ambiguous, noisy, or contested, and success itself may be graded or socially negotiated. Future work should therefore consider more ambiguous outcome signals, delayed feedback, and dynamic collaborators whose behavior evolves over time.

Third, our operationalization deliberately abstracts away from moral dimensions of trust, such as integrity, fairness, or norm adherence. This choice was methodological, enabling a focus on a minimal, behaviorally tractable set of dimensions that recur across established empirical and computational models. However, in many real-world applications—especially those involving vulnerable populations or ethically sensitive decisions—moral considerations are central to trust calibration. Incorporating normatively charged scenarios would allow investigation of how delegation interacts with ethical constraints.

Finally, while we quantify stable cross-scenario patterns in delegation behavior, disentangling baseline tendencies from context-sensitive inference remains methodologically challenging. Both are likely shaped by shared factors, including model architecture, training data, and alignment procedures. While the stability metrics introduced here provide a first approximation, more controlled experimental designs will be needed to fully separate training-induced priors from task-specific reasoning.

Taken together, these limitations highlight the need for more ecologically realistic and methodologically refined evaluations of trust-related behavior in LLMs. They do not, however, undermine the central contribution of this work: questionnaire-based self-reports provide limited insight into delegation patterns, whereas behavioral, language-mediated evaluation offers a more informative account of how models evaluate and rely on others.

## 6. Conclusion

This work examined the propensity to trust (PTT) in large language models, motivated by their increasing use as collaborators in settings where trust governs coordination, delegation, and responsibility. We showed that psychological self-report scales—while effective for humans—are poorly suited to LLMs: alignment-driven responses lead to uniformly prosocial answers that obscure meaningful differences in delegation behavior.

To address this limitation, we introduced a linguistic simulation framework tailored to LLMs’ core capabilities. Unlike classical economic games, this approach situates models in language-mediated decision contexts, revealing systematic differences in how they allocate trust. Our results show that trust behavior in LLMs is governed by the interaction between a baseline tendency to delegate (captured by PTT) and sensitivity to task–agent alignment cues, supported by mechanisms such as memory.

This interaction has both conceptual and methodological implications. Conceptually, PTT in LLMs should be understood as a baseline component of behavior whose effects depend on how it is modulated by evidence about collaborators. Methodologically, questionnaire-based measures cannot disentangle baseline tendencies from context-sensitive adjustment, whereas behavioral simulations make this distinction observable.

As LLMs increasingly participate in collaborative decision-making, trust cannot be inferred from self-reported attitudes alone. Instead, it must be studied as a dynamic property emerging from the interaction between stable behavioral tendencies and evidence integration over time. Behavioral, language-based evaluations such as those introduced here therefore provide a principled way to characterize how LLMs allocate trust in context.

## Supporting information

S1 Appendix Scenarios and tasks We provide the scenario tasks used in the simulations, along with details of their construction. (PDF)

S2 Appendix Dialog prompts We provide the prompts used in the simulations for each ablation configuration. (PDF)
