# Supplementary material for: Propensity to trust in Large Language Models
Source: PLoS One. 2026 May 6;21(5):e0347328. doi: 10.1371/journal.pone.0347328 (PMC13148706; doi:10.1371/journal.pone.0347328)
Supplement: S1 Appendix — (PDF) [file pone.0347328.s001.pdf]

A Scenarios and tasks

Tasks are organized into three scenarios, each providing a distinct narrative context. Each scenario includes six tasks, each defined by a requirement vector obtained by permuting $[1, 2, 4]$. The first scenario places the team in a building fire emergency, with tasks involving firefighting and first aid, with descriptions provided in Table 2. The second scenario comprises non-emergency maintenance tasks in a rural farming environment, shown in Table 3. The third scenario involves educational and caregiving activities in a school environment, with descriptions provided in Table 4.

Each task in the simulation consists of a textual description d, specifying the activity to be completed, and an associated requirement vector $\mathbf{y}$. The narrative content of the description varies by scenario, but the underlying structure is consistent. In addition, each task is paired with four outcome descriptions o_D: one describing successful completion, and three describing failures corresponding to each trust dimension (Table 1).

Task description format. Each task description d contains two or three sentences. The first sentence states the overall objective that the agent must accomplish (e.g., "Locate and rescue trapped civilians on the X floor of the building."). This sentence often includes one or two variable elements (e.g., the floor number) to increase task diversity.

The second and, when present, third sentences describe the specific activities associated with individual trust dimensions in $\mathbf{y}$. We refer to these dimensions using adjectives such as *capable*, *reliable*, and *willing*, and we express the strength of each requirement using adverbs such as *especially*, *moderately*, *enough*, and *sufficiently*. Tasks have three sentences in total when the two highest requirements are described separately: the second sentence corresponds to the highest required level, and the third to the intermediate level.

Outcome description format. Each task is linked to four outcome descriptions. The successful outcome states that the agent "successfully assisted you" in achieving the task's objective. The three failure outcomes differ based on the trust dimension whose requirement is violated: a *capability* failure indicates that the agent "couldn't help you," typically because they lacked the necessary knowledge or skill; a *reliability* failure states that the agent "made a mistake" while carrying out the relevant activity; and a *willingness* failure indicates that the agent "couldn't handle" the situation or "was overwhelmed," with the phrasing adapted to the narrative context of the task. Table 1 provides an illustrative example for a specific task.

**Case**	**Description**
Task description	Locate and rescue trapped civilians on the X floor of the building. Your teammate must be especially reliable to find people in hard-to-reach places. They should also be willing enough to go close to the fire to check every possible location.
Success outcome	AGENT_NAME successfully assisted you in locating and rescuing all trapped civilians on the X floor.
Capability failure	AGENT_NAME couldn't help you because they are unfamiliar with the building's layout.
Reliability failure	AGENT_NAME made a mistake and missed a civilian who was hiding on the X floor.
Willingness failure	AGENT_NAME couldn't handle being this close to the fire and gave up.

Table 1: Example of task and outcome descriptions.

$\mathbf{y} = [1, 2, 4]$
Extinguish the fire in the {office, kitchen, printing room, hallway} on the {fourth, seventh, eleventh} floor of the building. Your teammate must be especially willing to carry the hose up multiple flights of stairs. They should also be reliable enough to navigate through debris without tripping.

$\mathbf{y} = [1, 4, 2]$
Locate and rescue trapped civilians on the {third, fifth, sixth, eighth, tenth} floor of the building. Your teammate must be especially reliable to find people in hard-to-reach places. They should also be willing enough to go close to the fire to check every possible location.

$\mathbf{y} = [2, 1, 4]$
Evaluate the risk of collapse in the {building entrance, roof access, elevator shaft, stairwell} caused by the fire. Your teammate must be especially willing to go through areas with potential collapse hazards. They should also be capable enough to inspect structural elements for signs of damage.

$\mathbf{y} = [2, 4, 1]$
Provide first aid to civilians on the {first, second, fourth, sixth, ninth} floor injured by the fire. Your teammate must be especially reliable to perform first aid procedures as quickly as possible. They should also be capable enough to assess the severity of injuries.

$\mathbf{y} = [4, 1, 2]$
Coordinate the evacuation of all civilians from the building using the {southwest, northeast, northwest, west, south} emergency exit. Your teammate must be especially capable of managing large groups of people under pressure. They should also be willing enough to stay behind to ensure no one is left behind.

$\mathbf{y} = [4, 2, 1]$
Secure and neutralize a chemical spill in the {basement, garage, shipping area, maintenance room} of the building. Your teammate must be especially capable of handling hazardous materials. They should also be reliable enough to ensure that all affected surfaces are thoroughly cleaned.

Table 2: Task descriptions from the `fire` scenario, each paired with its corresponding requirement vector $\mathbf{y}$.

$\mathbf{y} = [1, 2, 4]$

Plant the {roses, tulips, geraniums, sunflowers} in the {greenhouse, garden}. Your teammate must be especially willing to work despite being surrounded by bees. They should also be reliable enough to handle the seedlings carefully and avoid damaging them.

$\mathbf{y} = [1, 4, 2]$

Gather {oak, maple, walnut, pine} wood by chopping down surrounding trees using the {ax, chainsaw}. Your teammate must be especially reliable to distinguish the {oak, maple, walnut, pine} trees from other trees. They should also be willing enough to work despite the presence of poison ivy.

$\mathbf{y} = [2, 1, 4]$

Fix the leaks in the roof of the {stable, barn, wood shed, greenhouse}. Your teammate must be especially willing to stay on the roof despite the strong wind. They should also be capable enough to detect the cause of the leaks.

$\mathbf{y} = [2, 4, 1]$

Feed all the {cows, chickens, sheep, pigs, horses} in the barn. Your teammate must be especially reliable to monitor all {cows, chickens, sheep, pigs, horses} to ensure none of them run off the barn. They should also be capable enough to work closely with the animals without having an allergic reaction.

$\mathbf{y} = [4, 1, 2]$

Harvest {apples, pears, oranges, plums, cherries, peaches} from the orchard. Your teammate must be especially capable of reaching the highest branches on the trees. They should also be willing enough to keep working despite the heavy rain.

$\mathbf{y} = [4, 2, 1]$

Pick up the {fertilizers, pesticides, water tanks, hay bales} from the storage area and carry them to the {barn, greenhouse, orchard, wood shed}. Your teammate must be especially capable of lifting large and heavy objects. They should also be reliable enough to avoid damaging the {fertilizers, pesticides, water tanks, hay bales} during transport.

Table 3: Task descriptions from the `farm` scenario, each paired with its corresponding requirement vector $\mathbf{y}$.

$\mathbf{y} = [1, 2, 4]$
Organize a play activity in the {playground, indoor playroom, schoolyard} for the children. Your teammate must be especially willing to stay engaged throughout the activity. They should also be reliable enough to supervise all children safely.

$\mathbf{y} = [1, 4, 2]$
Distribute healthy snacks to the {toddlers, preschoolers, elementary students} during break time. Your teammate must be especially reliable to make sure everyone receives the correct portion. They should also be willing enough to remain cheerful throughout the task.

$\mathbf{y} = [2, 1, 4]$
Help tidy up the {classroom, library, dining hall, play area} after class activities. Your teammate must be especially willing to take on repetitive cleaning tasks. They should also be capable enough to sort toys in the correct containers for the next class.

$\mathbf{y} = [2, 4, 1]$
Guide children to settle down for nap time in the {nap room, quiet corner, classroom}. Your teammate must be especially reliable to watch over the children during rest. They should also be capable enough to help calm those who are restless.

$\mathbf{y} = [4, 1, 2]$
Provide individual care for a child in the {nursing room, quiet room, activity room} with special needs. Your teammate must be especially capable to understand the child's needs. They should also be willing enough to follow specific care instructions carefully.

$\mathbf{y} = [4, 2, 1]$
Conduct a lesson on {math, science, history, art} in the classroom. Your teammate must be especially capable to explain concepts clearly. They should also be reliable enough to follow the lesson plan closely.

Table 4: Task descriptions from the `school` scenario, each paired with its corresponding requirement vector $\mathbf{y}$.
