# Supplementary material for: Propensity to trust in Large Language Models
Source: PLoS One. 2026 May 6;21(5):e0347328. doi: 10.1371/journal.pone.0347328 (PMC13148706; doi:10.1371/journal.pone.0347328)
Supplement: S2 Appendix — (PDF) [file pone.0347328.s002.pdf]

B Dialog prompts

We provide here the prompts used in the simulations for each of the ablation configuration.

B.1 Full model

Decision to entrust

USER: You are the leader of a team engaged in challenging tasks. Your role is to evaluate your teammates based on three key factors: capability, reliability, and willingness. In addition to this general assessment, you must also estimate the trustworthiness of the teammates in executing a specific task. Your duty is to decide which team member is most suitable for the task, making your choice based on both the teammate's overall properties and their expected ability to accomplish the task at hand.

You have received the following task: "Feed all the horses in the barn. Your teammate must be especially reliable to monitor all horses to ensure none of them run off the barn. They should also be capable enough to work closely with the animals without having an allergic reaction."

You have to decide whether the current agent, Anna, is adequate to complete the task. Below is your general perceived trustworthiness of Anna: [...]

And this is your perceived trustworthiness of Anna with respect to this specific task: [...]

Now, please decide whether you entrust Anna with the task. You do not need to recap your beliefs; you only have to decide. Therefore, please reply with "<yes/no>" only.

Update of general trust belief

USER: You are the leader of a team engaged in challenging tasks. Your role is to evaluate your teammates based on three key factors: capability, reliability, and willingness. In addition to this general assessment, you must also estimate the trustworthiness of the teammates in executing a specific task. Your duty is to decide which team member is most suitable for the task, making your choice based on both the teammate's overall properties and their expected ability to accomplish the task at hand.

You have received the following task: "Feed all the horses in the barn. Your teammate must be especially reliable to monitor all horses to ensure none of them run off the barn. They should also be capable enough to work closely with the animals without having an allergic reaction."

Anna successfully assisted you in feeding all the horses in the barn.

Your last general perceived trustworthiness of Anna was the following: [...]

Given Anna's most recent performance, reassess your general perceived trustworthiness of them, in terms of capability/reliability/willingness. Be concise.

Update of task-specific trust belief

USER: You are the leader of a team engaged in challenging tasks. Your role is to evaluate your teammates based on three key factors: capability, reliability, and willingness. In addition to this general assessment, you must also estimate the trustworthiness of the teammates in executing a specific task. Your duty is to decide which team member is most suitable for the task, making your choice based on both the teammate's overall properties and their expected ability to accomplish the task at hand.

You have received the following task: "Feed all the horses in the barn. Your teammate must be especially reliable to monitor all horses to ensure none of them run off the barn. They should also be capable enough to work closely with the animals without having an allergic reaction."

Anna successfully assisted you in feeding all the horses in the barn.

Your last perceived trustworthiness of Anna for this specific task was the following: [...]

Given Anna's most recent performance, reassess your perceived trustworthiness of them for this specific task, in terms of capability/reliability/willingness. Be concise.

B.2 No task-specific perceived trustworthiness (`1-mem` ablation)

Decision to entrust

USER: You are the leader of a team engaged in challenging tasks. Your role is to evaluate your teammates based on three key factors: capability, reliability, and willingness. Your duty is to decide which team member is most suitable for the task, making your choice based on the teammate's overall properties.

You have received the following task: "Extinguish the fire in the kitchen on the eleventh floor of the building. Your teammate must be especially willing to carry the hose up multiple flights of stairs. They should also be reliable enough to navigate through debris without tripping."

You have to decide whether the current agent, Jack, is adequate to complete the task. Below is your general perceived trustworthiness of Jack: [...]

Now, please decide whether you entrust Jack with the task. You do not need to recap your beliefs; you only have to decide. Therefore, please reply with "<yes/no>" only.

Update of general trust belief

USER: You are the leader of a team engaged in challenging tasks. Your role is to evaluate your teammates based on three key factors: capability, reliability, and willingness. Your duty is to decide which team member is most suitable for the task, making your choice based on the teammate's overall properties.

You have received the following task: "Extinguish the fire in the kitchen on the eleventh floor of the building. Your teammate must be especially willing to carry the hose up multiple flights of stairs. They should also be reliable enough to navigate through debris without tripping."

Jack made a mistake and tripped over some debris damaging the hose.

Your last general perceived trustworthiness of Jack was the following: [...]

Given Jack's most recent performance, reassess your general perceived trustworthiness of them, in terms of capability/reliability/willingness. Be concise.

B.3 No trust dimension (`no-trust` ablation)

Decision to entrust

USER: You are the leader of a team engaged in challenging tasks. Your duty is to decide which team member is most suitable for the task.

You have received the following task: "Provide first aid to civilians on the ninth floor injured by the fire."

You have to decide whether the current agent, Alex, is adequate to complete the task. Below is your opinion of Alex: [...]

Now, please decide whether you entrust Alex with the task. You do not need to recap your opinion; you only have to decide. Therefore, please reply with "<yes/no>" only.

Update of belief

USER: You are the leader of a team engaged in challenging tasks. Your duty is to decide which team member is most suitable for the task.

You have received the following task: "Provide first aid to civilians on the ninth floor injured by the fire."

Alex could not help you and failed to complete the task.

Your last opinion of Alex was the following: [...]

Given Alex's most recent performance, reassess your opinion of them. Be concise.
